# Lineage-specific early complete donor chimerism and risk of relapse after allogeneic hematopoietic stem cell transplantation for acute myeloid leukemia

**DOI:** 10.1038/s41409-022-01615-8

**Published:** 2022-02-24

**Authors:** Hannes Lindahl, Sofie Vonlanthen, Davide Valentini, Andreas T. Björklund, Mikael Sundin, Stephan Mielke, Dan Hauzenberger

**Affiliations:** 1 Clinical Immunology and Transfusion Medicine, Karolinska University Hospital, Stockholm, Sweden; 2 Department of Clinical Neuroscience, Karolinska Institutet, Stockholm, Sweden; 3 Department of Cellular Therapy and Allogeneic Stem Cell Transplantation (CAST), Karolinska University Hospital, Stockholm, Sweden; 4 Pediatric Hematology, Immunology and HCT, Astrid Lindgren Children’s Hospital, Karolinska University Hospital, Stockholm, Sweden; 5 Pediatrics, CLINTEC, Karolinska Institutet, Stockholm, Sweden; 6 Department of Cell Therapy and Allogeneic Stem Cell Transplantation (CAST), Department of Laboratory Medicine (LabMED), Karolinska University Hospital and Institutet, Karolinska Comprehensive Cancer Center, Stockholm, Sweden

**Keywords:** Allotransplantation, Prognosis, Risk factors, Acute myeloid leukaemia

## Abstract

Recipient–donor chimerism is routinely analyzed after allogeneic hematopoietic stem cell transplantation (HSCT) to monitor engraftment and graft rejection. For malignancies, chimerism can also be used to screen for disease relapse post-HSCT but methodology and interpretation of results are not standardized and likely depend on underlying diagnosis. We have implemented highly sensitive and accurate methodologies for chimerism analysis for the purpose of improving relapse prediction. Here, we report an exploratory retrospective analysis of clinical routine chimerism results from all 154 HSCTs for acute myeloid leukemia (AML) performed at our center during the years 2015–2020 with the aim of suggesting a clinically useful threshold at which risk of relapse is high. Relapse was not reliably predicted based on single elevated chimerism values obtained before time of overt relapse. However, early complete donor chimerism, here defined as recipient DNA < 0.2% in CD33^+^ cells in any blood or bone marrow sample taken during the first 60 days after HSCT, correlated inversely with relapse during the observation time (log-rank test *P* = 0.033). We propose that achievement of complete chimerism determined early after HSCT using sensitive methods can be used for risk-stratification of AML patients.

## Introduction

Allogeneic hematopoietic stem cell transplantation (HSCT) is a potentially curative treatment for hematological malignancies. Acute myeloid leukemia (AML) is the most common form of leukemia in adults and is a common indication for allogeneic HSCT [1]. Three-year overall survival after transplantation varies depending on AML risk profile but is on average less than 60% in Europe [2, 3]. The prognosis after a post-HSCT AML relapse has improved somewhat over time but still remains poor [4]. Impending relapse may respond to treatment, primarily using donor lymphocyte infusion (DLI) or tapering of immunosuppression and in certain cases chemotherapy, second transplantation, or immune therapy [5, 6]. Immune therapies currently under investigation are expected to further increase the treatment options [7, 8]. Consequently, there is a large clinical need for improved early detection of relapse after allogeneic HSCT.

Mixed recipient–donor chimerism has repeatedly been associated with relapse of AML after allogeneic HSCT in both adult and pediatric patients, but how the individual values should be interpreted in clinical practice remains unclear [9]. Ideally, monitoring of relapse after allogeneic HSCT should be done as frequently as is practical to identify and treat potential relapses as early as possible. Relapse prediction after allogeneic HSCT is currently performed either by detection of minimal residual disease (MRD), using flow cytometry or real-time quantitative polymerase chain reaction (qPCR), or by estimating the proportion of recipient DNA i.e. chimerism [10]. AML is genetically heterogeneous and detection of tumor specific MRD markers is often not possible, and markers may be lost as disease evolves potentially leading to relapse in MRD marker-negative patients [11, 12]. Whereas MRD analysis is mainly performed on bone marrow samples, chimerism is readily applied to blood samples making frequent sampling more feasible. Inherent to the method, chimerism is only applicable in the context of allogeneic transplantations but can be used in essentially all leukemia subtypes. Apart from relapse, chimerism is also used to assess engraftment and graft rejection, which is a benefit but also complicates interpretation.

Chimerism analysis by short tandem repeats (STR)-PCR is established as the gold standard [13, 14]. Although sensitivity is limited to 1–5% recipient DNA, increasing values has been associated with risk of relapse [15]. Chimerism-guided intervention has shown promising results of increased overall survival [9]. To achieve higher sensitivity, qPCR-based methods were developed [16] and studies indicate better performance in predicting relapse using serial testing in peripheral blood [17]. However, qPCR lack precision throughout the range of detection [18, 19]. We and others therefore used the two methods in parallel for some time. More recently, next generation sequencing (NGS) assays have been developed, which combine the sensitivity of qPCR assays and the precision of STR-PCR [19, 20]. Even greater sensitivity can potentially be achieved by determining chimerism values for each of the major cell types separately by sorting cells prior to DNA extraction, as recipient DNA may not be detectable in all cell types leading to dilution of the signal. There are reports suggesting that even low absolute values of chimerism or subtle increments over time may predict relapse [17, 21]. For this reason, we argue that a method, or combination of methods, that achieves both high sensitivity and accuracy is required to harness the full potential of chimerism analysis for relapse prediction.

Here, we report retrospective data on all allogeneic HSCTs for AML at Karolinska University Hospital in Sweden during the years 2015–2020 with the aim of suggesting a clinically useful threshold at which the risk of relapse motivates increased monitoring of the individual patient and potentially also preemptive interventions.

## Methods

### Patients and HSCTs

This study was approved by the Swedish Ethical Review Authority and was conducted in accordance with the Declaration of Helsinki. Informed consent was obtained from all included patients. All consecutive HCSTs for AML performed at Karolinska University Hospital from 2005 until the end of 2020 were included. Clinical data incorporated in the European Society for Blood and Marrow Transplantation (EBMT) registry were used. Patient and transplant characteristics are presented in Table 1. Subclassification of included AML diagnoses are presented in table S1. The conditioning regimens varied and have been categorized as myeloablative conditioning and reduced intensity conditioning according to established criteria [22].

**Table 1:** Patient and transplantation characteristics.

	All	With relapse	No relapse	*P*
Transplants	154	37 (24)	117 (76)	
Age at HSCT in y, median(range)	51 (4–73)	50 (5–73)	49 (4–73)	NS
Male	92 (60)	20 (54)	72 (62)	NS
Type of conditioning				
Myeloablative	108 (71)	26 (70)	82 (70)	NS
Reduced intensity	45 (29)	10 (27)	35 (30)	NS
Donor type				
Identical sibling	41 (28)	12 (32)	29 (25)	NS
Haploidentical related	11 (7)	–	11 (9)	NS
Matched unrelated	102 (68)	25 (68)	77 (66)	NS
Stem cell source				
Peripheral blood	135 (88)	33 (89)	102 (87)	NS
Bone marrow	19 (12)	4 (11)	15 (13)	NS
Patients with DLI treatment post-HSCT	11 (7)	8 (22)	3 (3)	<0.0001
All cause mortality	35 (23)	28 (76)	7 (6)	<0.00001
Days to relapse, median (range)	–	175 (29–817)	–	–
Days of follow-up, median (range)	1138 (99–2233)	1413 (281–2206)	1029 (99–2233)	0.016

Unless otherwise indicated, data are shown as *n* (%)*.*

*P* values for comparisons of transplants with relapse and without relapse are calculated using the χ2 test or unpaired students *T*-test as applicable.

*NS* Not statistically significant, *HSCT* Hematopoietic stem cell transplantation, *DLI* Donor lymphocyte infusion.

### Cell separation

Cells were routinely separated into the major cell types using Dynabeads (ThermoFisher, Waltham, MA, USA) coupled to antibodies, generating separate results for bone marrow cells expressing CD3, CD19, CD33, or CD34 and in the case of blood, for cells expressing CD3, CD19, or CD33. For routine separation, 4 ml of blood is processed but for small children as little as 2 ml has been successfully used. For bone marrow, 3 ml sample volume is requested but as little as 1 ml has been processed successfully. Samples are generally processed within 24 h but can be stored up to 48 h at 4 °C before proceeding with the cell separation. Phosphate buffered saline (PBS) is added to a total volume of 5 ml. In the Arrow robot, 30 µl of washed beads are added followed by a 15-min incubation. Beads are then separated using a magnet and the negative fraction is used for subsequent separations. For blood samples, the cell separations proceed in the following order: CD19, CD3, and CD33. For bone marrow samples, the original sample is split in two of which one is used for CD19 and CD3 and the other for CD34 and CD33 separation (in that order). The positive fractions are washed at least three times in PBS before proceeding with cell lysis and DNA extraction. The purity of cell separations is not routinely assessed but has been tested during the validation of the methods. The purity for blood samples was generally >95% and for bone marrow samples 70–90%.

### Chimerism

The majority of the chimerism results were generated by STR-PCR quantification using capillary electrophoresis or qPCR. Both methods included prior screening of recipient and donor DNA to find informative markers, for which one (STR-PCR) or two (qPCR) were used for chimerism analysis of post-HSCT samples. The qPCR method was based on Taqman probes (ThermoFisher) of single nucleotide polymorphisms (SNP) or indels at 24 different loci. Routinely, STR-PCR was chosen for samples with higher amounts of mixed chimerism, taking advantage of the method’s good accuracy, and qPCR was chosen for samples with low amounts of mixed chimerism, taking advantage of the method’s good sensitivity [18]. In 2020, these two methods were replaced by a NGS-based method (Devyser AB, Stockholm, Sweden), which has both good accuracy throughout the analytical range and good sensitivity [19]. Chimerism is reported as percentage of recipient DNA throughout. Samples that did not generate a reliable chimerism result due to technical issues were removed in this study. Bone marrow and blood samples for recipient chimerism analysis were taken at the clinician’s discretion and sampling frequencies are not uniform (Table 2). Consequently, patients were excluded when chimerism results were unavailable at time points and/or from sample types of interest for the analyses.

**Table 2:** Chimerism data.

	Bone marrow		Blood	
	relapse	No relapse	relapse	No relapse
Samples per patient	3 (2–7)	6 (4–8)	3 (2–4)	2 (1–4)
Patients with chimerism data, *n* (%)	33 (89)	102 (90)	34 (92)	103 (91)
Sampling interval before relapse, days	50 (30–67)	–	52 (31–113)	–

Unless otherwise indicated data are shown as median (IQR).

*IQR* Interquartile range.

### Statistical methods

Descriptive statistics of patient characteristics and HSCT regimens are reported for the combined data set as well as separately for those that had a relapse and those that had no relapse during the study time. Four patients had their first and second HSCT within the study time making the total number of patients in the study fewer than the total number of transplantations. However, for all statistical models each transplantation is treated equally. Groups were compared using the χ^2^ test for categorical data and unpaired students *t*-test for numerical data. Simple and multiple logistic regression were used to assess the relationship between relapse and chimerism values as well as other independent variables that were considered potential confounders. Receiver Operating Characteristics (ROC) curve analysis was used to determine discriminative performance of the models and the optimal cutoff was determined using the Youden’s J statistic (Youden’s index). Relapse-free survival was calculated with the Kaplan–Meier method and the log-rank test was used for comparisons of curves. Correlations for non-normally distributed data were calculated using Spearman’s rank correlation coefficient. *P* values are two sided and 0.05 was considered statistically significant. All calculations and figures were performed using R v 3.6.1 (the Comprehensive R Archive Network project) with RStudio v. 1.1.456 and the packages ggplot2, ROCit, and survminer.

## Results

### Patients and samples

A total of 154 HSCTs were performed for 150 patients with AML during 2015–2020 (Table 1). Included AML subtypes are listed in table S1. The study includes both pediatric (*n* = 10) and adult patients (*n* = 140) and the median age was 51 years (range, 4–73 years). The median follow-up time was 1,138 days (range 99–2,233). Thirty-seven transplants (24%) resulted in a relapse within the study time after a median of 75 days post-HSCT. No statistically significant association between relapse and age, sex, type of conditioning, donor type, or stem cell source was observed (Table 1). Relapse was however strongly associated with death. All available chimerism data for these patients were extracted, in total 5009 values from 1399 unique biological samples (Table 2). Whenever a bone marrow and blood sample were taken on the same day for chimerism analysis, these were considered as paired samples and the correlation between obtained values from these samples were plotted (Fig. S1).

### Mixed chimerism and prediction of relapse

**Fig. 1:**
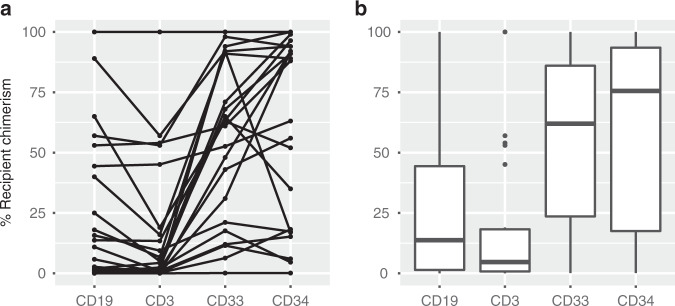
Chimerism results from bone marrow samples taken at relapse. Percentage of recipient chimerism in four sorted cell fractions from bone marrow samples taken at the time of hematological relapse shown as (**a**) dots where individual patients are connected by lines and (**b**) boxplots representing the median and interquartile range.

**Fig. 2:**
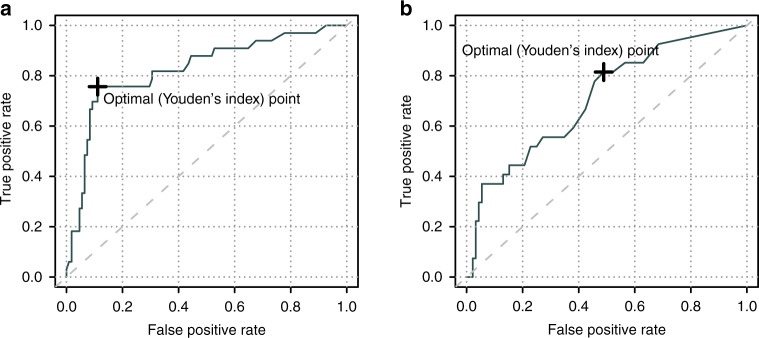
Discrimination power of single chimerism values in CD33^+^ cells to predict impending or overt relapse. Receiver operating characteristics (ROC) curves depicting the true positive rate (sensitivity) and false positive rate (1-specificity) of relapse prediction in acute myeloid leukemia patients after hematopoietic stem cell transplantation. Chimerism from (**a**) bone marrow and (**b**) blood samples were analyzed at the clinician’s discretion. The analysis includes chimerism values obtained from 30 days after transplantation to the day of clinical relapse or an equal length of time in the group that did not have a relapse. Youden’s index suggest a cutoff that makes a trade-off between sensitivity and specificity.

First, we assessed how well individual chimerism values can predict relapse if anticipation is not required. Chimerism results were filtered to remove samples that (1) were taken during the first 30 days after HSCT because mixed chimerism during this time most likely reflects engraftment (2) were taken after relapse or (3) were taken in the non-relapse group later than the average time to relapse in the relapse group, to adjust the observation time in the two groups. The variable %_max_ was defined as the highest chimerism value for each transplantation during this time window (30 to on average 485 days after HSCT). 619 bone marrow samples from 141 transplants, of which 33 led to a relapse, were available. Thirteen transplants were thus excluded because no bone marrow sample for chimerism was taken during this time window. Univariable analysis showed a statistically significant association between relapse and %_max_ recipient chimerism in bone marrow samples, which was most pronounced in CD33^+^ cells (Table 3). Multivariable analysis showed that this association was independent of other plausible predictive factors of relapse. At relapse, CD34^+^ and CD33^+^ cell chimerism were essentially correlated but a subset of patients had distinctly higher chimerism in CD34^+^ cells (Fig. 1). For %_max_ in bone marrow CD33^+^ cells, receiver-operating characteristics (ROC)-analysis resulted in an area under the curve (AUC) of 0.83 (Fig. 2). Youden’s index suggested an optimal cutoff at 6.3%, which resulted in a sensitivity of 76% and a specificity of 89%. The same analysis was then performed using the chimerism values from blood samples. After removing transplants without blood chimerism values (*n* = 20), 134 remained of which 26 led to a relapse. In contrast to bone marrow, univariable analysis did not result in a statistically significant association between %_max_ in blood samples and relapse in any of the three sorted cell populations (Table 3). However, the number of blood samples were only 273 compared to 619 bone marrow samples, reflecting the practice at our center.

**Table 3:** Relapse predicted by max recipient chimerism (%_max_) in samples taken before or at time of relapse.

Univariable	Bone marrow (*n* = 141)			Blood (*n* = 119)		
	*P*	OR	CI 95%	*P*	OR	CI 95%
CD3	0.009	1.03	1.01–1.06	0.18	1.03	0.99–1.07
CD19	0.003	1.03	1.01–1.05	0.70	1.01	0.95–1.06
CD33	2.3 × 10^−6^	1.04	1.02–1.05	0.24	1.02	0.99–1.06
CD34	3.3 × 10^−6^	1.03	1.02–1.04	–	–	–
**Multivariable**						
CD33	2.6 × 10^−6^	1.04	1.02–1.06	0.21	1.02	0.99–1.06
Patient age	0.82	1.00	0.97–1.04	0.95	1.00	0.97–1.03
CR vs not	0.23	0.41	0.09–1.89	0.05	0.28	0.07–1.06
MAC vs RIC	0.68	1.30	0.36–4.68	0.75	1.22	0.35–4.30
Related vs not	0.57	1.34	0.48–3.67	0.92	1.05	0.39–2.77

*P* values are from logistic regression analysis.

*OR* Odds ratio, *CI* Confidence interval, *CR* Complete remission before transplantation, *MAC* Myeloablative conditioning, *RIC* Reduced intensity conditioning, *Related vs not* HLA-identical sibling or haploidentical relative vs matched unrelated donor, *n* number of transplants.

To assess whether individual chimerism values in this data set can predict relapse in advance, thus enabling preemptive measures to restrain the tumor cells, we excluded chimerism values at the time of overt relapse. In patients that did not have a relapse we excluded values that were taken later than the mean time from HSCT to last sample before overt relapse in the relapse group. Again, samples from the first 30 days after HSCT were removed. For the analysis of bone marrow samples, this left 503 chimerism values from 134 transplants of which 26 led to a relapse and for blood samples, 232 chimerism values from 118 transplants of which 26 led to a relapse. However, no statistically significant association between relapse and %_max_ in any sample or cell type was observed (Table S2). The median number of samples per patient that remained after the filtration steps were four and two for bone marrow and blood, respectively.

### Early complete donor chimerism and prediction of relapse

**Fig. 3:**
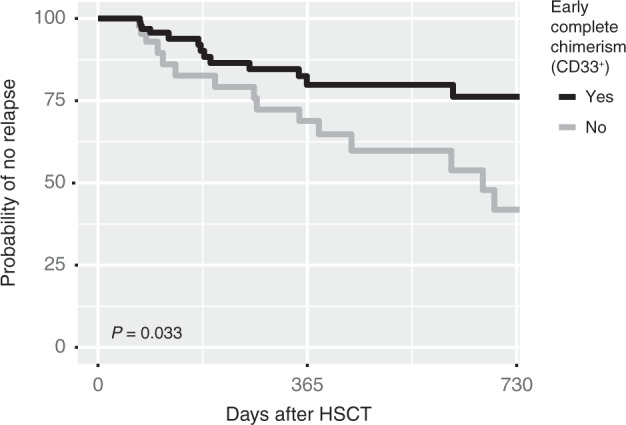
Complete chimerism within the first 60 days after HSCT is an early indicator of relapse risk for patients with acute myeloid leukemia. The lowest chimerism value in CD33^+^ cells from blood or bone marrow sampled during the first 60 days after hematopoietic stem cell transplantation (HSCT) was used to stratify patients based on if early complete chimerism was achieved, here defined as having <0,2% recipient DNA at any time during the first 60 days after HSCT. The two groups were compared using Kaplan–Meier estimates regarding relapse-free disease course.

Next, we investigated if low recipient chimerism values early after HSCT predicts a relapse-free disease course aiming to gain prognostic information related to the graft rather than to detect recurring disease. Analogous to previous analyses we defined the variable %_min_ as the lowest chimerism value obtained during the first 60 days after HSCT. Bone marrow sampling during this time after HSCT is infrequent at our center. We therefore first analyzed only chimerism values from blood samples and then combined all values from both compartments for an additional analysis. For blood samples, univariable and multivariable analyses demonstrated a statistically significant association between future relapse and %_min_ in CD33^+^ cells (Table S3). Similar results were obtained if chimerism values from both blood and bone marrow were included, implying that results from both compartments are informative for the purpose of predicting future relapse. We continued with the combined chimerism values from CD33^+^ blood and bone marrow cells and performed a ROC-curve analysis. The discriminative efficacy for %_min_ to predict future relapse was modest, with an AUC of 0.63. Youden’s index suggested an optimal cutoff of 0.2%, which resulted in a sensitivity of 50% and a specificity of 75%. Next, we investigated if early complete chimerism using this low threshold of recipient DNA may be a useful means to stratify patients as having high or low risk of relapse. We dichotomized all patients based on if early complete chimerism was reached, defined as at least one chimerism value below 0.2% in either blood or bone marrow CD33^+^ cells in the first 60 days after HSCT. Patients that had a relapse within the first 60 days after HSCT were excluded from this analysis (*n* = 2). 141 Patients of which 30 had a relapse were included. Early complete chimerism was observed in 50% of patients that later had a relapse and 75% of patients that did not have a relapse during the study time (χ^2^ *P* = 0.017). Univariable and multivariable analysis was performed and showed a statistically significant association between early complete chimerism and future relapse (Table 4). Kaplan–Meier analysis showed a statistically significant lower probability of relapse in patients with early complete donor chimerism (log-rank test *P* = 0.033) (Fig. 3). The probability of being relapse-free two years after HSCT for patients that achieved early complete chimerism was estimated at 0.76 (95% CI 0.65–0.90) compared to 0.42 (0.25–0.70). The same Kaplan–Meier analysis performed on only blood (*n* = 135) or bone marrow (*n* = 44) samples showed similar trends but did not reach statistical significance (Fig. S2).

**Table 4:** Relapse predicted by complete donor chimerism (<0.2% recipient) in samples taken the first 60 days after HSCT.

Univariable	Bone marrow or blood (*n* = 141)			Blood (*n* = 136)		
	*P*	OR	CI 95%	*P*	OR	CI 95%
CD3	0.41	0.71	0.30–1.60	0.51	0.78	0.33–1.81
CD19	0.30	1.83	0.63–6.70	0.51	1.44	0.52–4.68
CD33	0.011	0.34	0.15–0.78	0.033	0.40	0.17–0.93
**Multivariable**						
CD33	0.008	0.29	0.11–0.72	0.017	0.32	0.12–0.81
Patient age	0.52	1.01	0.98–1.05	0.46	1.01	0.98–1.05
CR vs not	0.18	0.40	0.10–1.69	0.21	0.42	0.11–1.80
MAC vs RIC	0.89	0.92	0.25–3.25	0.89	0.91	0.25–3.22
Related vs not	0.29	0.59	0.21–1.52	0.32	0.61	0.21–1.58

*P* values are from logistic regression analysis.

*OR* Odds ratio, *CI* Confidence interval, *CR* Complete remission before transplantation, *MAC* Myeloablative conditioning, *RIC* Reduced intensity conditioning, *Related vs not* HLA-identical sibling or haploidentical relative vs matched unrelated donor, *n* Number of transplants.

## Discussion

Relapse is the most common cause of death after allogeneic HSCT for AML [23] but treatments exist that may prevent a clinical relapse in its early stages [5–8]. Impending relapse can potentially be detected by tumor specific MRD markers or chimerism analysis. Chimerism has repeatedly been associated with relapse risk but cutoff values have not been established [9, 24]. Our initial aim was to investigate in our retrospective data if increased recipient chimerism could be an early biomarker of an impending relapse and to suggest a cutoff value at which the risk is high. However, relapses in adults with AML often occur very rapidly and with the sampling frequency performed in clinical practice at our center, it was not possible to predict relapse with a sufficient degree of anticipation. Previous studies have suggested that more frequent chimerism analysis is required [25, 26]. On the other hand, the data showed that failing to achieve early complete donor chimerism, using a very stringent threshold of <0.2% recipient DNA in CD33^+^ cells during the first 60 days after HSCT, was associated with increased risk of relapse and consequently has potential to be used for risk-stratification of AML patients post-HSCT.

Interestingly, chimerism in bone marrow and blood do not correlate reliably in our data (Fig. S1), which also has been observed by others [27–29] suggesting that the two compartments reflect different processes or have different kinetics. The observation that chimerism in bone marrow samples associated more strongly with relapse in our data than chimerism in peripheral blood samples may have a biological explanation and/or may be a consequence of less frequent sampling from peripheral blood. Monitoring chimerism in peripheral blood cells would be preferable because more frequent sampling is possible but based on our data the utility of peripheral blood chimerism analyses is difficult to assess and further elucidation of this would require a different study design.

At our center, we routinely perform chimerism analysis on fractionated cells to (1) achieve greater sensitivity and (2) add information about the graft composition. It has been shown that higher donor T cell chimerism in peripheral blood samples taken early after transplantation is associated with better prognosis [30]. In our data, we observed no statistically significant association between mixed chimerism in T cells and relapse. However, relapse was most consistently associated with mixed chimerism in the CD33^+^ fraction of bone marrow samples, reflecting the myeloid origin of the tumor cells. Chimerism in CD34^+^ was distinctly elevated in a large portion of patients at the time of overt relapse (Fig. 1), which may indicate a leukemic stem cell phenotype [31].

Donor chimerism gradually increases during the first days to weeks after allogeneic HSCT reflecting engraftment and graft alloreactivity. Various definitions of complete donor chimerism have been used, for example <5–10% recipient. In most cases complete chimerism occurs within 30 days after HSCT [32, 33]. Previous retrospective studies of the utility of chimerism in AML patients post-HSCT have suggested that relapse can be predicted based on whether complete chimerism is achieved or not. One study defined complete chimerism as recipient DNA < 1% at any point in time after HSCT and only 4/75 patients failed to achieve this, of which three relapsed [34]. The largest retrospective study to date of the prognostic value of chimerism included relapse prediction as a secondary outcome [35] and included 193 AML patients. With a threshold of 10% recipient DNA, significant associations between chimerism in unsorted cells around day 30 as well as around day 100 with subsequent relapse were observed. In that study, chimerism was assessed by STR-PCR, which is less sensitive than qPCR or NGS and values from bone marrow and blood samples were analyzed together. Chimerism in CD3^+^ cells was analyzed in parallel but no added benefit from this was observed. Our data similarly suggest that assessment of complete chimerism early after allogeneic HSCT for AML has potential as a prognostic marker. Remaining recipient-derived cells in either blood or bone marrow samples during this time window could serve as an indicator of the alloreactivity of the graft and thus efficiency of the graft vs leukemia effect. The association between low chimerism (%_min_) early post-HSCT and reduced risk of subsequent relapse was limited to the CD33^+^ fraction. This may reflect presence of remaining myeloid leukemia cells that have acquired a survival benefit compared to other recipient cells. Both qPCR and NGS are sensitive methods that reliably quantify chimerism >0.01 and >0.1, respectively [19]. With sensitive methods a low threshold can be applied that may increase the predictive properties of early complete donor chimerism.

In summary, achievement of complete donor chimerism assessed by sensitive methods early after HSCT for AML appears to be useful for relapse risk-stratification. The data suggest that patients that do not reach a stringently defined threshold of complete donor chimerism in blood or bone marrow samples during the first two months after HSCT should be monitored more closely. With the sampling frequency used at our center, mixed chimerism does not detect a developing relapse in its early stages but can confirm a manifested relapse. Questions that remain are whether mixed chimerism can be used to guide preemptive measure in the early stages of a relapse and whether analysis of blood samples has advantages over bone marrow samples because of the possibility of higher sampling frequencies. This should preferably be addressed in prospective studies.

## Supplementary information

Supplementary
