# Supplementary material for: Lineage-specific early complete donor chimerism and risk of relapse after allogeneic hematopoietic stem cell transplantation for acute myeloid leukemia
Source: Bone Marrow Transplant. 2022 Feb 24;57(5):753–9. doi: 10.1038/s41409-022-01615-8 (PMC9090632; doi:10.1038/s41409-022-01615-8)
Supplement: Supplementary file 1 — Supplementary [file 41409_2022_1615_MOESM1_ESM.docx]

**Supplementary Information**

*Lineage-specific early complete donor chimerism and risk of relapse after allogeneic hematopoietic stem cell transplantation for acute myeloid leukemia.*

*Lindahl et al.*

| **Table S1. AML subtypes** | |
| --- | --- |
|  |  |
|  | n |
| AML w recurrent genetic abnormalities | 2 |
| AML w t(8;21)(q22;q22); RUNX1-RUNX1T1 | 1 |
| AML with inv(16)(p13.1q22) or t(16;16)(p13.1;q22); CBFB-MYH11 | 3 |
| Acute promyelocytic leukaemia with t(15;17)(q22;q12); PML-RARA | 1 |
| AML with t(9;11)(p22;q23); MLLT 3-MLL | 3 |
| AML with t(6;9)(p23;q24); DEK-NUP214 | 2 |
| AML with inv(3)(q21q26.2) or t(3;3)(q21;q26.2); RPN1-EVI1 | 2 |
| AML w MDS type changes (incl AML transf from MDS or overlap MDS/MPN) | 26 |
| AML with minimal differentiation [M0] | 7 |
| AML without maturation [M1] | 3 |
| AML with maturation [M2] | 5 |
| Acute myelomonocytic leukemia [M4] | 6 |
| Acute monoblastic and monocytic leukemia [M5] | 11 |
| Acute erythroid leukemia [M6] | 2 |
| Myeloid sarcoma (Granulocytic sarcoma) | 2 |
| Blastic plasmacytoid dendritic cell neoplasm (BPDCN) | 2 |
| Therapy related myeloid neoplasm (old Secondary AML) | 6 |
| AML with 11q23 (MLL) abnormalities | 1 |
| AML with mutated NPM1 | 9 |
| AML with biallelic mutation of CEBPA | 1 |
| AML with mutated RUNX1 | 8 |
| Other AML | 41 |
| Unknown | 10 |

| **Table S2. Relapse predicted by maximum recipient chimerism (%_max_) in samples taken before time of relapse** | | | | | | | |
| --- | --- | --- | --- | --- | --- | --- | --- |
|  | **Bone marrow** (n = 134) | | |  | **Blood** (n = 118) | | |
| *Univariable* | *P* | OR | CI 95% |  | *P* | OR | CI 95% |
| CD3 | 0.24 | 1.02 | 0.98-1.05 |  | 0.23 | 1.02 | 0.98-1.07 |
| CD19 | 0.72 | 0.99 | 0.92-1.03 |  | 0.92 | 1.00 | 0.89-1.05 |
| CD33 | 0.75 | 1.00 | 0.97-1.03 |  | 0.31 | 1.02 | 0.98-1.06 |
| CD34 | 0.33 | 1.01 | 0.99-1.02 |  | - | - | - |
|  |  |  |  |  |  |  |  |
| *Multivariable* |  |  |  |  |  |  |  |
| CD33 | 0.98 | 1.00 | 0.97-1.03 |  | 0.27 | 1.02 | 0.98-1.06 |
| Patient age | 0.27 | 1.02 | 0.99-1.06 |  | 0.96 | 1.00 | 0.97-1.03 |
| CR vs not | 0.09 | 0.30 | 0.08-1.33 |  | 0.05 | 0.27 | 0.07-1.03 |
| MAC vs RIC | 0.51 | 1.58 | 0.40-6.32 |  | 0.89 | 1.09 | 0.30-3.91 |
| Related vs not | 0.84 | 0.90 | 0.32-1.06 |  | 0.86 | 1.09 | 0.40-2.90 |
| *P*-values are from logistic regression analysis. OR, odds ratio, CI, confidence interval; CR, complete remission before transplantation; MAC, myeloablative conditioning; RIC, reduced intensity conditioning; Related vs not, HLA-identical sibling or haploidentical relative vs matched unrelated donor; n, number of transplants. | | | | | | | |

| **Table S3. Relapse predicted by minimum donor chimerism (%_min_) in samples taken the first 60 days after HSCT** | | | | | | | |
| --- | --- | --- | --- | --- | --- | --- | --- |
|  | **Blood** (n = 136) | | |  | **Bone marrow or blood** (n = 141) | | |
| *Univariable* | *P* | OR | CI 95% |  | *P* | OR | CI 95% |
| CD3 | 0.21 | 1.14 | 0.97-1.14 |  | 0.045 | 1.11 | 1.0-1.24 |
| CD19 | 0.50 | 0.72 | 0.17-1.26 |  | 0.36 | 0.53 | 0.07-0.94 |
| CD33 | 0.04 | 1.20 | 1.03-1.49 |  | 0.049 | 1.18 | 1.01-1.44 |
|  |  |  |  |  |  |  |  |
| *Multivariable* |  |  |  |  |  |  |  |
| CD33 | 0.029 | 1.22 | 1.04-1.52 |  | 0.04 | 1.20 | 1.02-1.48 |
| Patient age | 0.37 | 1.02 | 0.98-1.05 |  | 0.40 | 1.01 | 0.98-1.05 |
| CR vs not | 0.15 | 0.38 | 0.10-1.57 |  | 0.19 | 0.41 | 0.12-1.70 |
| MAC vs RIC | 0.86 | 1.12 | 0.32-3.91 |  | 0.84 | 1.13 | 0.32-4.00 |
| Related vs not | 0.68 | 0.82 | 0.31-2.04 |  | 0.66 | 0.81 | 0.31-2.00 |
| *P*-values are from logistic regression analysis. OR, odds ratio, CI, confidence interval; CR, complete remission before transplantation; MAC, myeloablative conditioning; RIC, reduced intensity conditioning; Related vs not, HLA-identical sibling or haploidentical relative vs matched unrelated donor; n, number of transplants. | | | | | | | |


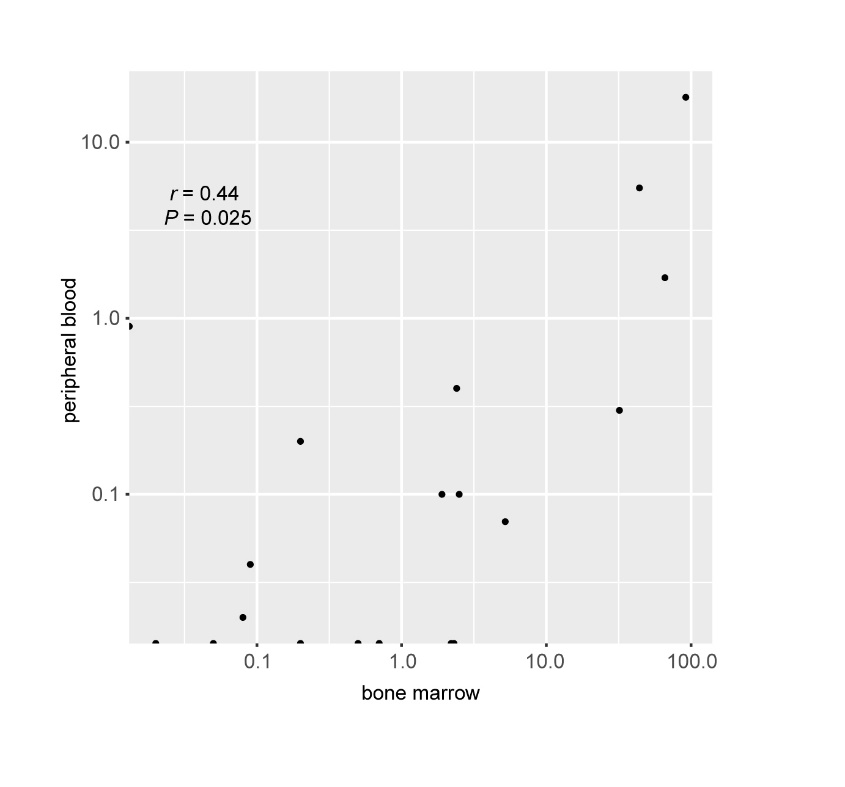


**Figure S1. All paired peripheral blood and bone marrow samples.** Percentage of recipient chimerism in CD33^+^ cells when peripheral blood and bone marrow were sampled on the same day from the same patient. Correlation was assessed using Spearman’s rank correlation coefficient *r*. *P*-value is two-sided.


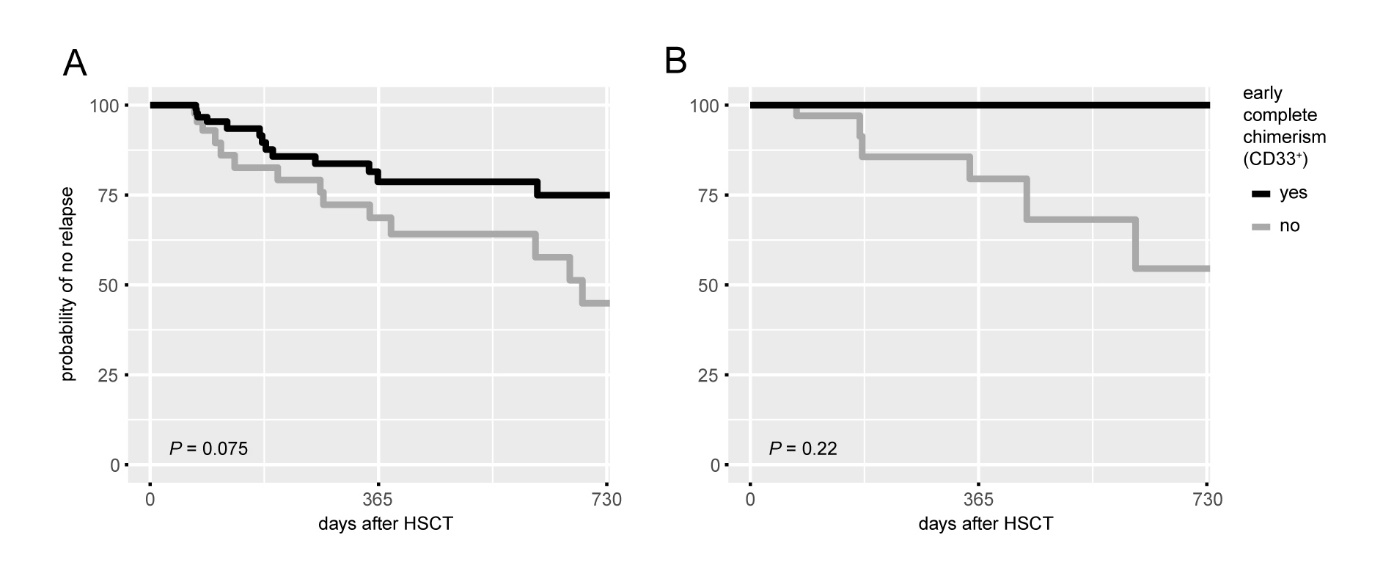


**Figure S2. Complete chimerism within the first 60 days after HSCT in blood and bone marrow samples** (A) The lowest chimerism value in CD33^+^ cells from (A) blood or (B) bone marrow sampled during the first 60 days after hematopoietic stem cell transplantation (HSCT) is used to stratify patients based on if early complete chimerism was achieved, here defined as having <0,2% recipient DNA at any time during the first 60 days after HSCT. The two groups were compared using Kaplan-Meier estimates regarding relapse-free disease course.
